# Trends in Participation in Medicare Among Psychiatrists and Psychiatric Mental Health Nurse Practitioners, 2013-2019

**DOI:** 10.1001/jamanetworkopen.2022.24368

**Published:** 2022-07-29

**Authors:** Seokmin Oh, Alex McDowell, Nicole M. Benson, Benjamin Lê Cook, Vicki Fung

**Affiliations:** 1 Harvard University, Cambridge, Massachusetts; 2 Mongan Institute, Massachusetts General Hospital, Boston; 3 Department of Medicine, Harvard Medical School, Boston, Massachusetts; 4 McLean Hospital, Belmont, Massachusetts; 5 Department of Psychiatry, Harvard Medical School, Boston, Massachusetts; 6 Health Equity Research Lab, Cambridge Health Alliance, Cambridge, Massachusetts

## Abstract

This cross-sectional study examines trends in the number of psychiatrists and psychiatric mental health nurse practitioners overall and for Medicare beneficiaries from 2013 to 2019.

## Introduction

Medicare beneficiaries are more likely than the general adult population to experience mental illness.^1^ However, insufficient numbers of psychiatric clinicians and limited insurance participation are associated with restricted availability of care.^2,3^ Increases in the number of psychiatric mental health nurse practitioners (PMHNPs) could help mitigate poor access to care, particularly for psychiatric prescribing. The primary care nurse practitioner workforce has increased in underserved rural areas^4^; however, data are limited on PMHNPs. We examined the number of psychiatrists and PMHNPs overall and for Medicare beneficiaries from 2013 to 2019.

## Methods

In this repeated cross-sectional study, we assessed the number of psychiatrists and PMHNPs with a National Provider Identifier from 2013 to 2019 using the National Plan & Provider Enumeration System (NPPES) file. We then examined the number and percentage who participated in Medicare, defined as billing for professional services or being the prescribing clinician on Part D claims for 11 or more Medicare beneficiaries per year using publicly available Medicare data.

We assessed the percentage of Hospital Service Areas (HSAs) in 2013 and 2019 with only Medicare psychiatrists, only Medicare PMHNPs, both, or neither. Results were stratified for rural (micropolitan to rural) vs urban (metropolitan) areas based on Rural-Urban Commuting Area codes.

Changes were assessed using linear probability models with HSA-level random effects and year fixed effects; differences between rural and urban HSAs were examined using Pearson χ^2^ tests. Details about data sources and definitions are available in eTable 1 and eTable 2 in the Supplement.

Analyses used a 2-sided *P* < .05 as a threshold for statistical significance and were conducted using Stata version 14.0 (StataCorp). This study was approved by the Mass General Brigham institutional review board with waiver of informed consent as secondary use of research data and followed the STROBE reporting guideline for cross-sectional studies.

## Results

Between 2013 and 2019, the number of psychiatrists in the NPPES file increased from 51 166 to 58 814 and the number of PMHNPs from 7132 to 16 698 (Table 1). Medicare participation rates decreased among psychiatrists from 60.7% to 55.1% and remained stable for PMHNPs at approximately 63.0%.

**Table 1.** Number of Psychiatrists and PMHNPs Who Billed or Prescribed for 11 or More Medicare Beneficiaries per Year From 2013 to 2019

Year	No. in NPPES file	No. (%)		
		Billed traditional Medicare for professional services	Part D prescriber	Billed Medicare for professional services or was a Part D prescriber
Psychiatrists				
2013	51 166	22 213 (43.4)	28 972 (56.6)	31 057 (60.7)
2014	52 370	22 324 (42.6)	29 430 (56.2)	31 492 (60.1)
2015	53 393	22 286 (41.7)	29 552 (55.3)	31 669 (59.3)
2016	54 570	22 374 (41.0)	29 523 (54.1)	31 814 (58.3)
2017	55 432	22 189 (40.0)	29 153 (52.6)	31 607 (57.0)
2018	57 131	22 278 (39.0)	29 358 (51.4)	31 944 (55.9)
2019	58 814	22 419 (38.1)	29 711 (50.5)	32 420 (55.1)
Change 2013-2019, %	14.9	0.9 (–12.2)	2.6 (–10.8)	4.4 (–9.2)
PMHNPs				
2013	7132	3179 (44.6)	4121 (57.8)	4455 (62.5)
2014	8123	3677 (45.3)	4775 (58.8)	5122 (63.1)
2015	9134	4232 (46.3)	5501 (60.2)	5844 (64.0)
2016	10 423	4842 (46.5)	6176 (59.3)	6605 (63.4)
2017	11 711	5488 (46.9)	7110 (60.7)	7539 (64.4)
2018	13 842	6397 (46.2)	8385 (60.6)	8863 (64.0)
2019	16 698	7505 (44.9)	9917 (59.4)	10 522 (63.0)
Change 2013-2019, %	134.1	136.1 (0.8)	140.6 (2.8)	136.2 (0.9)

Abbreviations: NPPES, National Plan & Provider Enumeration System, PMHNP, psychiatric mental health nurse practitioner.

Of 3436 HSAs, the percentage with no Medicare psychiatrists or Medicare PMHNPs decreased from 39.8% in 2013 to 36.8% in 2019 (Table 2). The percentage of HSAs with only Medicare psychiatrists decreased from 26.2% to 14.0%. The percentage with both Medicare psychiatrists and PMHNPs increased from 30.3% to 41.9% and with only PMHNPs from 3.7% to 7.4% (*P* < .001 for all comparisons).

**Table 2.** Changes in the Availability of Psychiatrists and PMHNPs Participating in Medicare Across HSAs Overall and for Rural and Urban HSAs, 2013-2019

Variable	No. (%)		Percentage change (95% CI)^a^
	2013	2019	
All HSAs (n = 3436)			
No Medicare psychiatrists or PMHNPs	1368 (39.8)	1263 (36.8)	–3.1 (–4.1 to –2.0)
Only Medicare PMHNPs	127 (3.7)	253 (7.4)	3.7 (2.8 to 4.6)
Only Medicare psychiatrists	899 (26.2)	481 (14.0)	–12.2 (–13.6 to –10.7)
Both Medicare psychiatrists and PMHNPs	1042 (30.3)	1439 (41.9)	11.6 (10.2 to 12.9)
Rural HSAs (n = 1437)			
No Medicare psychiatrists or PMHNPs	896 (62.4)	857 (59.6)	–2.7 (–4.6 to –0.9)
Only Medicare PMHNPs	81 (5.6)	156 (10.9)	5.2 (3.6 to 6.8)
Only Medicare psychiatrists	277 (19.3)	178 (12.4)	–6.9 (–8.8 to –5.0)
Both Medicare psychiatrists and PMHNPs	183 (12.7)	246 (17.1)	4.4 (2.8 to 5.9)
Urban HSAs (n = 1999)			
No Medicare psychiatrists or PMHNPs	472 (23.6)	406 (20.3)	−3.3 (–4.6 to –2.0)
Only Medicare PMHNPs	46 (2.3)	97 (4.9)	2.6 (1.6 to 3.6)
Only Medicare psychiatrists	622 (31.1)	303 (15.2)	−16.0 (–18.0 to –13.9)
Both Medicare psychiatrists and PMHNPs	859 (43.0)	1193 (59.7)	16.7 (14.8 to 18.6)

Abbreviations: HSAs, hospital service areas; PMHNPs, psychiatric mental health nurse practitioners.

^a^ To assess changes in 2019 vs 2013 in the percentage of HSAs with PMHNP and/or psychiatrists overall and for rural and urban areas, we used linear probability models with HSA-level random effects and year fixed effects. We used Pearson χ^2^ tests to assess differences in the proportion of rural vs urban HSAs with PMHNPs and/or psychiatrists in each year. Excludes less than 0.3% of psychiatrists or PMHNPs in the National Plan & Provider Enumeration System file in each year whose addresses did not link with an HSA.

In 2013 and 2019, rural HSAs were more likely than urban HSAs to have no Medicare psychiatrists or PMHNPs (for 2019, 59.6% vs 20.3%; *P* < .001). Rural vs urban HSAs were also more likely to have only Medicare PMHNPs (for 2019, 10.9% vs 4.9%; *P* < .001) but less likely to have Medicare psychiatrists only or in combination with PMHNPs.

## Discussion

Although Medicare participation rates among psychiatrists decreased between 2013 and 2019, the number of PMHNPs providing care to Medicare beneficiaries more than doubled. Most rural HSAs had no Medicare psychiatrists or PMHNPs.^5^ Rural HSAs were less likely than urban HSAs to have Medicare psychiatrists in 2013 and 2019, although increases in the proportion of HSAs with PMHNPs may have helped some rural areas maintain access to psychiatric prescribers.

A limitation is that this study excluded clinical nurse specialists and psychologists who can prescribe in some states. We may have underestimated PMHNP participation in Medicare due to the prevalence of incident-to-billing under which care provided by PMHNPs is billed under a physician’s National Provider Identifier number.^6^ Identification of care delivery models that maximize the availability of different clinician types is critical for improving access to mental health care, particularly in rural areas.

## Notes

Supplement. **eTable 1.** Description of Data Sources and Definitions **eTable 2.** Taxonomy Codes Used to Identify Psychiatrists and PMHNPs

## References

[1] Figueroa JF, Phelan J, Orav EJ, Patel V, Jha AK. Association of mental health disorders with health care spending in the Medicare population. JAMA Netw Open. 2020;3(3):e201210-e201210. doi:10.1001/jamanetworkopen.2020.1210 32191329 PMC7082719

[2] Benson NM, Myong C, Newhouse JP, Fung V, Hsu J. Psychiatrist participation in private health insurance markets: paucity in the land of plenty. Psychiatr Serv. 2020;71(12):1232-1238. doi:10.1176/appi.ps.202000022 32811283 PMC7708395

[3] Bishop TF, Press MJ, Keyhani S, Pincus HA. Acceptance of insurance by psychiatrists and the implications for access to mental health care. JAMA Psychiatry. 2014;71(2):176-181. doi:10.1001/jamapsychiatry.2013.2862 24337499 PMC3967759

[4] Xue Y, Smith JA, Spetz J. Primary care nurse practitioners and physicians in low-income and rural areas, 2010-2016. JAMA. 2019;321(1):102-105. doi:10.1001/jama.2018.17944 30620363 PMC6583766

[5] Smith AS, Trevelyan E. The older population in rural America: 2012-2016. American Community Survey Reports. September 2019. Accessed June 23, 2022. https://www.census.gov/content/dam/Census/library/publications/2019/acs/acs-41.pdf

[6] Medicare Payment Advisory Commission. Medicare and the health care delivery system. June 14, 2019. Accessed June 23, 2022. https://www.medpac.gov/wp-content/uploads/import_data/scrape_files/docs/default-source/reports/jun19_medpac_reporttocongress_sec.pdf

