# Supplementary material for: Trends in Participation in Medicare Among Psychiatrists and Psychiatric Mental Health Nurse Practitioners, 2013-2019
Source: JAMA Netw Open. 2022 Jul 29;5(7):e2224368. doi: 10.1001/jamanetworkopen.2022.24368 (PMC9338410; doi:10.1001/jamanetworkopen.2022.24368)
Supplement: Supplement. — eTable 1. Description of Data Sources and Definitions eTable 2. Taxonomy Codes Used to Identify Psychiatrists and PMHNPs [file jamanetwopen-e2224368-s001.pdf]

Supplemental Online Content

Oh S, McDowell A, Benson NM, Cook BL, Fung V. Trends in participation in Medicare among psychiatrists and psychiatric mental health nurse practitioners, 2013-2019. *JAMA Netw Open.* 2022;5(7):e2224368.
doi:10.1001/jamanetworkopen.2022.24368

eTable 1. Description of Data Sources and Definitions

eTable 2. Taxonomy Codes Used to Identify Psychiatrists and PMHNPs

This supplemental material has been provided by the authors to give readers additional information about their work.

© 2022 Oh S et al. *JAMA Network Open*.

eTable 1: Description of data sources and definitions

Data source	Description
National Bureau of Economic Research (NBER) National Plan & Provider Enumeration System (NPPES) File, 2013-2019	Used to identify the total number of psychiatrists and PMHNPs with active National Provider Identifiers in each year based on taxonomy codes and provider practice address ZIP codes for linkage with Hospital Service Areas
Centers for Medicare & Medicaid Services (CMS) Physician and Other Supplier File, 2013-2019	Used to identify the total number of psychiatrists and PMHNPs who billed for professional services for 11 or more unique Medicare beneficiaries each year. This public use file is based on 100% of Medicare Part B non-institutional claims. Limited to fee-for-service Traditional Medicare.
CMS Part D Prescriber File, 2013-2019	Used to identify the total number of psychiatrists and PMHNPs who prescribed drugs for 11 or more unique Medicare beneficiaries each year. This public use file is based on 100% Medicare Part D Event files, which include both Traditional Medicare and Medicare Advantage beneficiaries.
Dartmouth Atlas Project, Hospital Service Area ZIP Code Crosswalks	Used to map psychiatrists and PMHNPs by practice address ZIP code to Hospital Service Areas
U.S. Department of Agriculture Rural-Urban Commuting Areas (RUCA) codes	Used to defined whether a Hospital Service Area is rural (RUCA codes 4-10) or urban (1-3). We assigned HSAs the highest urbanicity code among their corresponding ZIP codes:<br>1. Metropolitan area core: primary flow within an urbanized area (UA)<br>2. Metropolitan area high community: primary flow 30% or more to a UA<br>3. Metropolitan area low commuting: primary flow 10% to 30% to a UA<br>4. Micropolitan area core: primary flow within an urban cluster of 10,000 to 49,999 (large UC)<br>5. Micropolitan high commuting: primary flow 30% or more to a large UC<br>6. Micropolitan low commuting: primary flow 10% to 30% to a large UC<br>7. Small town core: primary flow within an urban cluster of 2,500 to 9,999 (small UC)<br>8. Small town high commuting: primary flow 30% or more to a small UC<br>9. Small town low commuting: primary flow 10% to 30% to a small UC<br>10. Rural areas: primary flow to a tract outside a UA or UC<br>99. Not coded: Census tract has zero population and no rural-urban identifier information

© 2022 Oh S et al. *JAMA Network Open*.

eTable 2: Taxonomy codes used to identify psychiatrists and PMHNPs

Taxonomy Code	Description
2084A0401X	Psychiatry & Neurology : Addiction Medicine
2084B0040X	Psychiatry & Neurology : Behavioral Neurology & Neuropsychiatry
2084F0202X	Psychiatry & Neurology : Forensic Psychiatry
2084H0002X	Psychiatry & Neurology : Hospice and Palliative Medicine
2084P0800X	Psychiatry & Neurology : Psychiatry
2084P0802X	Psychiatry & Neurology : Addiction Psychiatry
2084P0804X	Psychiatry & Neurology : Child & Adolescent Psychiatry
2084P0805X	Psychiatry & Neurology : Geriatric Psychiatry
2084P2900X	Psychiatry & Neurology : Pain Medicine
2084S0012X	Psychiatry & Neurology : Sleep Medicine
363LP0808X	Nurse Practitioner: Psychiatric/Mental Health

© 2022 Oh S et al. *JAMA Network Open*.
